# Green Tea Polyphenol Epigallocatechin-Gallate in Amyloid Aggregation and Neurodegenerative Diseases

**DOI:** 10.3389/fnins.2021.718188

**Published:** 2021-09-14

**Authors:** Luiza Fernandes, Thyago R. Cardim-Pires, Debora Foguel, Fernando L. Palhano

**Affiliations:** Instituto de Bioquímica Médica Leopoldo de Meis, Programa de Biologia Estrutural, Universidade Federal do Rio de Janeiro, Rio de Janeiro, Brazil

**Keywords:** amyloidosis, epigallocatechin-gallate, anti-amyloidogenic, Alzheimer’s disease, Parkinson’s disease, Huntington’s disease

## Abstract

The accumulation of protein aggregates in human tissues is a hallmark of more than 40 diseases called amyloidoses. In seven of these disorders, the aggregation is associated with neurodegenerative processes in the central nervous system such as Alzheimer’s disease (AD), Parkinson’s disease (PD), and Huntington’s disease (HD). The aggregation occurs when certain soluble proteins lose their physiological function and become toxic amyloid species. The amyloid assembly consists of protein filament interactions, which can form fibrillar structures rich in β-sheets. Despite the frequent incidence of these diseases among the elderly, the available treatments are limited and at best palliative, and new therapeutic approaches are needed. Among the many natural compounds that have been evaluated for their ability to prevent or delay the amyloidogenic process is epigallocatechin-3-gallate (EGCG), an abundant and potent polyphenolic molecule present in green tea that has extensive biological activity. There is evidence for EGCG’s ability to inhibit the aggregation of α-synuclein, amyloid-β, and huntingtin proteins, respectively associated with PD, AD, and HD. It prevents fibrillogenesis (*in vitro* and *in vivo*), reduces amyloid cytotoxicity, and remodels fibrils to form non-toxic amorphous species that lack seed propagation. Although it is an antioxidant, EGCG in an oxidized state can promote fibrils’ remodeling through formation of Schiff bases and crosslinking the fibrils. Moreover, microparticles to drug delivery were synthesized from oxidized EGCG and loaded with a second anti-amyloidogenic molecule, obtaining a synergistic therapeutic effect. Here, we describe several pre-clinical and clinical studies involving EGCG and neurodegenerative diseases and their related mechanisms.

## Protein Aggregation and Amyloid Diseases (AmD)

Amyloid fibrils are proteinaceous, insoluble structures that can be formed and accumulated inside or outside cells in response to mutations, stress conditions (pH, temperature, ionic strength, etc.), increase in protein concentration, and cellular protein quality-control failure, among others (Husby and Sletten, 1986; Brange et al., 1992; Chi et al., 2003; Alford et al., 2008). The vast majority of amyloid fibrils is composed of cross-beta structure and is found in different organs and tissues, causing a heterogeneous group of intractable diseases collectively called amyloid diseases (AmD) or amyloidoses (Husby and Sletten, 1986). The common structural features of all amyloid fibrils have allowed the use of specific probes such as Congo red (reviewed by Yakupova et al., 2019) and thioflavin-T (Biancalana and Koide, 2010) to evaluate their formation in the test tube or in the diagnosis of AmD. Universal antibodies against fibrils have also been developed (Kayed et al., 2007). This homogeneity in fibril structure has been exploited in the search for anti-amyloidogenic compounds with some success, at least in the test tube (Trivella et al., 2012; Sant’anna et al., 2013). Epigallocatechin-gallate (EGCG) is one of these successful examples, as described in this review.

Amyloid diseases can be either systemic or localized. In the former, amyloid deposits are mainly found dispersed among peripheral tissues/organs, while in the latter the aggregates are restricted to a specific tissue/organ; if they occur in the central nervous system (CNS) a neurodegenerative disorder can develop (Muchowski, 2002; Wechalekar et al., 2016), as in the case of Huntington’s disease (HD), Parkinson’s disease (PD), and Alzheimer’s disease (AD) (DiFiglia et al., 1997; Rocha et al., 2018; Busche and Hyman, 2020). Although they all share the amyloid fibrils as a hallmark, the proteins that compose the fibrils and the regions of the brain where the deposits are found, at least at the onset of each disease, are different, which gives rise to different clinical manifestations and demands the use of different palliative treatments, since up to now, there are no drugs against these diseases (Rubinsztein, 2006; Bloom, 2014). Tafamidis is an exception since it has been used with great success in several countries in recent years to treat patients with familial amyloid polyneuropathy, a transthyretin (TTR)-related amyloidosis (Coelho et al., 2012), and more recently, familial cardiomyopathy (Maurer et al., 2018). TTR is a tetrameric protein with two thyroxin binding pockets in the dimer-dimer interface. This structural feature allowed the development of compounds (such as Tafamidis and tolcapone) that fit with high affinity into these pockets, locking TTR in its tetrameric, non-amyloidogenic state. Tafamidis is now commercially available. This fortunate circumstance has not occurred with other amyloidogenic proteins, some of which even belong to the family of intrinsically disordered proteins, which makes it very difficult to find compounds that trap these proteins in a non-aggregating conformation. Thus, strategies or compounds that target very early aggregate species, blocking the progress of aggregation, are lacking.

Nowadays, there is a consensus that most of the toxicity/damage observed in AmD is due mainly to the oligomeric, soluble species that are formed in the process of fibril formation (Verma et al., 2015). In addition, amyloid fibrils can serve as a reservoir of toxic, soluble oligomers contributing to disease progression (Azevedo et al., 2012). Despite their different protein origins, oligomers share morphologies and biological activities (Kayed and Lasagna-Reeves, 2013). Their toxicity is associated with their binding to different cellular receptors, their pore-forming capacity, and their ability to modulate different cell pathways, among other properties (Chong et al., 2006; Choi et al., 2013; Miller et al., 2014). In the case of neurodegenerative AmD, dysregulation of synapses (pre- or post-synaptic neurons) (Scheff et al., 2007; Koffie et al., 2009; da Silva et al., 2020; Marcantoni et al., 2020), induction of reactive oxygen species (ROS) and oxidative stress (Figueiredo et al., 2013; Deas et al., 2016), calcium imbalance and mitochondrial dysfunction (Luth et al., 2014; Ludtmann et al., 2018), apoptosis induction (Chong et al., 2006), cell membrane adhesion and toxicity (Choi et al., 2013; Burré et al., 2014) and other cellular effects have been observed.

Amyloid diseases are multifactorial diseases since in addition to the damage caused by protein deposition *per se* and oligomer-related injuries, inflammation also plays an important role in disease progression and prognosis (Azevedo et al., 2019; La Vitola et al., 2021). Promising compounds must cross the blood–brain barrier (BBB) when neurodegenerative disorders are considered. All this complexity has to be considered in the search for new therapeutic approaches, which are urgent in an aging population.

## Natural Products as Therapeutic Molecules: Flavonoids of Green Tea

The use of natural therapeutic approaches was described more than 3,000 years ago, mostly by Chinese and Indian medicine (Nestler, 2002; Saini, 2016). The secondary metabolites from plants represent an endless frontier to be explored in the search for compounds with pharmaceutical and medical purpose. We know only a tiny fraction of our biodiversity worldwide, which makes our responsibility to preserve the environment very important.

Secondary metabolism provides several groups of molecules with different chemical properties and biochemical activities such as alkaloids, tannins, quinones, saponins, methylxanthines, and flavonoids (Kumar and Pandey, 2013). Flavonoids consist of molecules of a phenolic nature that share the flavone group as the primary skeleton. Its complexity allows different substitutions in the structure, leading to formation of a diversity of compounds such as rutin, quercetin, hesperidin, and epigallocatechin-3-gallate (Manach et al., 2004; Wen et al., 2017). The diversity of these molecules is vital to the plants, since they act as antioxidant and vegetal hormones to protect them from ultraviolet rays, bugs and opportunistic microorganisms (Wen et al., 2017).

Flavonoids display multiple biological properties such as antioxidation, through direct interaction with ROS; anticancer, by modulating several cellular pathways involved in tumor growth and apoptosis, anti-inflammation, and other effects (de Almeida et al., 2020; de Amorim et al., 2020; Lakshmi et al., 2020; Xuan et al., 2020).

Tea, the second most consumed beverage in the world, is rich in flavonoids. The predominant flavonoids of tea are catechins: (–)-epicatechin, (–)-epicatechin gallate, (–)-epigallocatechin, and (–)-epigallocatechin gallate. Tea also contains other phenolic acids (e.g., gallic acid), minerals (e.g., potassium and calcium), and amino acids (e.g., theanine) that contribute to its nutraceutical properties (Balentine et al., 1997).

Different types of tea (e.g., black and green) are prepared from the leaves of *Camellia sinensis*, whose production and consumption are widespread in China (Yan et al., 2020). The dry weight of a fresh leaf contains 2.5 to 4% caffeine and 30% flavonoids (Balentine et al., 1997).

**FIGURE 1:**
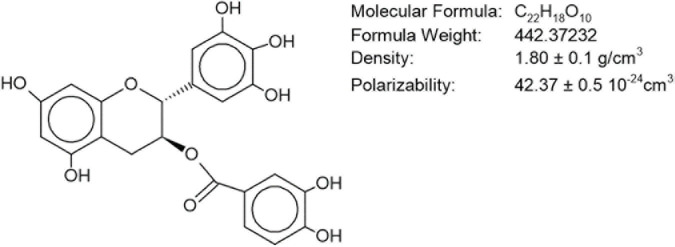
Molecular structure of epigallocatechin-gallate.

The flavonoid EGCG is a polyphenolic compound having seven hydroxyl radicals distributed among three aromatic rings (Figure 1). This feature confers water solubility, allowing this molecule to be extracted merely by boiling in water (Wang et al., 2008).

Epigallocatechin-gallate oxidation can form a quinone state that can self-polymerize and crosslink with amines and sulfhydryl groups of proteins (Palhano et al., 2013). The quinone state is also related to amyloid fibril remodeling and conserves its anti-amyloidogenic activity (Palhano et al., 2013; An et al., 2017).

## History of the Inhibitory Effects of Epigallocatechin-Gallate in Protein Aggregation and Its Use in Neurodegenerative Amyloid Diseases

### Epigallocatechin-Gallate in Prion Diseases

The prion diseases are characterized by the conversion of the cellular isoform of the prion protein (PrP^*C*^) to scrapie (PrP^Sc^), a pathogenic conformation (reviewed by Prusiner et al., 1998; Colby and Prusiner, 2011). In this process, some of the α-helices present in PrP^C^ are refolded into β-sheets, which changes the protein physicochemical characteristics and leads to the formation of proteinaceous infectious particles, PrP^*Sc*^ (Pan et al., 1993; Prusiner et al., 1998). High-throughput screenings were performed to find effective therapeutic agents that could cross the BBB as well as inhibit the formation of PrP^Sc^; reduce the PrP^C^ precursor of infectious PrP^*Sc*^; and disaggregate pre-existing PrP^Sc^ (reviewed by Giles et al., 2017). In the screening of 2,000 drugs and natural products, some polyphenols such as tannin, 2′2′″-bisepigallocatechin digallate, and katacine were identified as anti-PrP^*Sc*^ agents (Kocisko et al., 2003). These compounds prevented PrP^Sc^ formation and accumulation in infected cells and inhibited the induced conversion of radiolabeled hamster PrP^C^ to PrP^Sc^, resistant to protease degradation (Kocisko et al., 2003). Other polyphenols, including epicatechin and epigallocatechin, were ineffective against PrP^Sc^ formation, indicating that structural variations can lead to opposite results (Kocisko et al., 2003). After cellular screening *in vitro* (Kocisko et al., 2003), some inhibitors such as polyphenolic extracts of tea, grape seed and pine bark, tannic acid, amodiaquine, thioridazine, thiothixene, trifluoperazine, and tetrandrine were tested in scrapie-infected mice, and the positive results observed previously did not occur *in vivo* (Kocisko et al., 2004). Multiple variables can be involved in this lack of an effect *in vivo*, such as dosage, vehicle, timing for beginning or ending treatment, the compound’s inability to cross the BBB and its metabolization (Kocisko et al., 2004).

A subsequent study specifically evaluated the effect of green tea extract on PrP^Sc^ formation (Rambold et al., 2008). It was demonstrated that EGCG and gallocatechin-gallate (GCG), abundant polyphenols in green tea, induced rapid transition of mature PrP^C^ into detergent-insoluble conformations, favoring the cellular protein internalization and its lysosomal degradation (Rambold et al., 2008). Consequently, PrP^Sc^ formation was reduced because the PrP^C^ precursor was depleted by the EGCG or GCG treatment (Rambold et al., 2008). Furthermore, the greater efficacy of EGCG compared to epicatechin-gallate (ECG) was attributed to the presence of three hydroxyl groups in the trihydroxyphenyl side chain and their meta-position in EGCG (Rambold et al., 2008). The gallate group at the side chain was described as essential for the observed anti-PrP^Sc^ effect (Rambold et al., 2008). Next, the effect of EGCG was also evaluated in the yeast prion protein Sup35 (Roberts et al., 2009). In these experiments, EGCG inhibited fibrillogenesis of Sup35’s prion domain *in vitro* at 25 or 37°C as measured by Congo red, ThT fluorescence, and sedimentation assays (Roberts et al., 2009), while epicatechin and gallic acid had no inhibitory effects (Roberts et al., 2009). Pre-formed fibers of Sup35 prion were incubated with EGCG and the drug promoted fibril remodeling after 24 h (Roberts et al., 2009). The treatment reduced the fibrillar content and promoted an enrichment of soluble oligomeric species (Roberts et al., 2009). These oligomers were not recognized by specific conformational antibodies for amyloid species and presented lower seeding capacity (Roberts et al., 2009). The yeast phenotype termed [PSI^+^] indicates a strain containing Sup35 in prion state aggregates and resistant to the action of proteases (Paushkin et al., 1997). The effect of EGCG on this phenotype was investigated, and the treatment reduced weak [PSI^+^] colonies (susceptible prion strain) more effectively than strong [PSI^+^] (resistant prion strain) (Roberts et al., 2009). Finally, EGCG and 4,5-bis- (4-methoxyanilino) phthalimide (DAPH-12) were co-applied to increase activity against prionogenesis, since DAPH-12 was able to cure strong colonies [PSI^+^] (Roberts et al., 2009). Therefore, the synergy with EGCG increased the elimination of several Sup35 prion strains and enhanced the polyphenol therapeutic potential (Roberts et al., 2009; Duennwald and Shorter, 2010).

The studies presented above have demonstrated the efficacy of EGCG in inhibiting prionogenesis (Kocisko et al., 2003; Rambold et al., 2008; Roberts et al., 2009), although the mechanism has not yet been clarified. Investigations using nuclear magnetic resonance (NMR) spectroscopy revealed that EGCG bound non-specifically with PrP^C^ (Kamatari et al., 2013). This bond promoted the structural stabilization of PrP^C^, interfering in the intermolecular interaction between PrP^C^ and PrP^Sc^ during the pathogenic conversion process (Kamatari et al., 2013). The neuroprotective action was also evaluated: neurotoxicity was induced by the PrP fragment (106–126) in human neuroblastoma cells and the consequences of EGCG pre-treatment were analyzed (Lee et al., 2015). When EGCG was present, it inhibited mitochondrial dysfunctions, preventing Bax translocation and cytochrome c release, and induced autophagy activated by sirt1 (silent mating type information regulation 2-homolog), increasing cell survival (Lee et al., 2015). Thus, these results suggest that EGCG may be useful for therapy in prion diseases.

### Effects of Epigallocatechin-Gallate in Huntingtin Aggregation

Huntington’s disease treatment is restricted to reducing symptoms (Kumar and Kumar, 2009; Dickey and La Spada, 2018; Varga et al., 2020) and the focus of research has been on disease-modifying treatments. For the first time, after the screening of 5,000 natural molecules, EGCG demonstrated potential modulatory effects against the early steps in huntingtin (htt) aggregation (Ehrnhoefer et al., 2006). A fluorescence microscopy assay revealed a reduction of protein aggregates by approximately 40% in a yeast model overexpressing htt (expanded with 72 glutamines and fused to green fluorescent protein), and the cell- extract evaluation confirmed the lower quantity of insoluble species (Ehrnhoefer et al., 2006). In yeast, EGCG decreased the toxicity promoted by htt and in HD transgenic flies the compound diminished the photoreceptor degeneration and motor impairments (Ehrnhoefer et al., 2006). Accordingly, the EGCG becomes attractive as an anti-amyloidogenic therapeutic strategy (Ehrnhoefer et al., 2006). Subsequently, EGCG was tested against fibrillar oligomers, which are globular structures, soluble in detergent and detected by an antibody that recognizes the fibrillar conformation of amyloid pathogenic proteins (Kumar and Kumar, 2009). The EGCG reduced these oligomers in pheochromocytoma cells (PC12) expressing htt and also reduced the formation of inclusion bodies (Kumar and Kumar, 2009). Given the complexity of amyloidoses and the influence of changes in the aggregation environment, the effectiveness of EGCG was also assessed in the presence of lipid membranes and the study demonstrated that EGCG efficacy was not impaired (Beasley et al., 2019). In addition to EGCG, the effect on HD of an infusion of green tea like that consumed by humans was analyzed using a *Drosophila* model. The green tea improved the neurodegeneration presented in HD flies but did not influence their viability or prolong the lifespan of wild-type *Drosophila* (Varga et al., 2020). The authors’ discussion highlights the modest positive effect of the tea infusion consumption on symptoms of HD and states that the results obtained can be limited by the genetic condition of *Drosophila*, the fly husbandry and the composition and concentration of green tea used for the test (Varga et al., 2020).

### Effects of EGCG on Amyloidogenesis of α-Synuclein Protein and Parkinson’s Disease Prevention

#### Effects on α-Synuclein Aggregation

The aggregation of α-synuclein (α-syn) is a process that occurs in PD (Cascella et al., 2021). Analysis of the therapeutic potential of EGCG have shown that it inhibits α-syn amyloidogenesis (Ehrnhoefer et al., 2008; Jha et al., 2017; Dominguez-Meijide et al., 2020) and protects rat PC12 and neuroblastoma cells against aggregate-induced cytotoxicity (Ehrnhoefer et al., 2008; Jha et al., 2017). Even with the presence of metal ions to accelerate the fibrillation process, EGCG has been shown to be an effective anti-amyloid agent (Zhao et al., 2017; Teng et al., 2019). The amyloid aggregation pathway was redirected to the formation of stable spherical oligomers when EGCG was added (Ehrnhoefer et al., 2008). These oligomers were identified as non-toxic amorphous species unable to promote seeding and disassembly under denaturing conditions (resistance to sodium dodecyl sulfate) (Ehrnhoefer et al., 2008). The presence of protein dimers, tetramers and hexamers was observed after treatment with EGCG, indicating crosslinking between α-syn and the compound (Ehrnhoefer et al., 2008). Moreover, it has been shown that EGCG binds specifically to intrinsically disordered proteins (α-syn and Aβ), preventing the conversion of random-coil structures into β-sheets (Ehrnhoefer et al., 2008). The EGCG interacted with flexible regions in natively structured proteins and natively disordered proteins (α-syn and Aβ) and the binding occurred uniformly throughout the protein sequence (Fusco et al., 2018). In addition, the protein oligomerization promoted by EGCG, which redirects the pathway of amyloid formation to amorphous species, probably occurs by the establishment of multiple hydrogen bonds and aromatic interaction with backbone atoms that inducing aggregation by protein-protein interaction (Fusco et al., 2018). It has been suggested that the aggregation process can be reduced by EGCG through the oxidation of α-syn methionines (Ponzini et al., 2019). A later study discriminated between the conformational states of α-syn in neutral pH solution in the absence and presence of EGCG, proposing that EGCG binds preferentially to compact α-syn species and does not depend on covalent modifications to establish the protein-ligand interaction (Konijnenberg et al., 2016).

Šneideris et al. (2015, 2019) argued that the EGCG may be not an inhibitor of amyloidogenesis and demonstrated the possibility of false-positive results related to the method of analysis applied and the influence of environmental conditions. When the ThT fluorescence assay, based on aggregation half-time, was used, Šneideris et al. (2015) observed that EGCG did not inhibit the formation of α-syn and Aβ fibrils. The same was observed when the pH was reduced from 7.0 to 6.0: the EGCG lost its anti-amyloid effectiveness (Šneideris et al., 2019). However, despite this controversy, several studies have reported the effectiveness of EGCG (Ehrnhoefer et al., 2008; Caruana et al., 2011; Jha et al., 2017; Zhao et al., 2017; Teng et al., 2019; Dominguez-Meijide et al., 2020).

The potential of EGCG was also evaluated in the inhibition of pre-formed amyloid fibrils (Bae et al., 2010; Bieschke et al., 2010; Yoshida et al., 2013; Haney et al., 2017; Jha et al., 2017). Similar to that described with α-syn monomers, new findings indicated that EGCG bound directly to β-sheets of fibrils, altering the amyloid conformation without disassembling them into toxic oligomeric intermediates (Bieschke et al., 2010). The remodeling of fibrils by the action of EGCG reduced the deposition of amyloid and transformed them into amorphous non-cytotoxic aggregates (Bieschke et al., 2010). Additionally, the effectiveness of EGCG was assessed in simulations of physiological conditions in a crowded macromolecular environment (Gautam et al., 2017). EGCG, in synergy with β-cyclodextrin, which also acts alone against aggregation (reviewed by Oliveri and Vecchio, 2016; Gautam et al., 2017), increased the inhibition of amyloidogenesis and the disaggregation of pre-formed fibrils (Gautam et al., 2017).

Based on the three-dimensional structure of the α-syn fibril, an analysis of the molecular dynamics of the atoms was performed to understand the remodeling process that occurs during the binding between EGCG and the mature fibril (Liu et al., 2018). The main types of EGCG interaction were hydrophobic and hydrogen bonding, affecting three different fibril sites and with participation to binding of some residues such as LYS58, GLU61, THR64, LYS96, and ASP98 (Liu et al., 2018). The remodeling promoted by EGCG occurred by generated disturbances in β-sheets and hydrogen bonds in turn of the structure of the peptide, disordering the fibril (Liu et al., 2018). It has been reported that EGCG reduced the ordered structure of the fibril (Liu et al., 2017; Yao et al., 2020) and enhanced the rupture of the β-sheets occurred mainly in the regions of residues 45–55 and 86–96, affecting the overall structure of the fibril (Yao et al., 2020). Furthermore, the EGCG interacted preferentially with the charged residues E46, E61, K80, and E83 and the polar residue S87 and with the hydrophobic residues H50, V66, V82, V95, and F94, besides destroying the saline bridge E46-K80, stabilizer of the amyloid structure (Yao et al., 2020). However, although these studies demonstrate the ability of EGCG to remodel fibrils, Sternke-Hoffmann et al. (2020) proposed that EGCG cannot inhibit its α-syn seeding capacity. Sternke-Hoffmann et al. (2020) argued that EGCG can interact with the fibril surface and block binding to ThT. Additionally, the conditions used during the tests, such as the types of plaques or pH of the solutions, alter the results of the remodeling of fibrils. It has been shown that, although EGCG promotes fibril remodeling, ThT may not be the best probe to assess the occurrence of this process (Kelley et al., 2021). Immediately after incubating the fibril with EGCG, a reduction in ThT fluorescence was observed, but this did not represent remodeling and when the washing protocol was applied, the free EGCG was removed and ThT levels were restored to a level similar to that observed prior to treatment (Kelley et al., 2021). Thus, the authors suggested the use of pentameric thiophene as an alternative to the use of ThT in addition to the application of complementary techniques and centrifugation/washing protocols to avoid unspecific results (Kelley et al., 2021).

#### Effects on Cellular Mechanism and Neuroprotection

The EGCG also proved to be an efficient amyloid antagonist when pre-formed oligomers were subjected to treatment (Caruana et al., 2011). These amyloid aggregation intermediates, soluble oligomers, can induce pore formation and permeabilization of the lipid bilayer, leading to cell death. Indeed, they are known as the most pathogenic amyloid species (Danzer et al., 2007). The treatment with EGCG was able to inhibit cytotoxicity induced by pre-fibrillar species in mouse neuroblastoma cells (Gautam et al., 2017). The inhibition promoted by EGCG may be related to its binding with the flexible C-terminal region of α-syn oligomers, reducing damage to the membrane (Lorenzen et al., 2014). The evidence points to a decrease in the oligomer-membrane interaction after treatment of vesicles with EGCG, which may be consequently associated to the protection of rat brain cells against oligomer toxicity (Lorenzen et al., 2014). Another proposition suggests that EGCG accelerates the formation of amyloid fibrils, reducing the active toxic oligomers (Yang et al., 2017). Thus, the cellular protection displayed after treatment with EGCG would be to facilitate the conversion of active oligomers into amyloid fibrils, decreasing rupture of the cell membrane and the cytotoxicity of the aggregates (Yang et al., 2017).

A different mechanism has been suggested for the action of EGCG in the yeast model of α-synucleinopathy (Griffioen et al., 2006). Due to its antioxidant and metal-chelating properties, EGCG inhibited aggregation and cytotoxicity. The polyphenol also preserved dopaminergic neurons and motor functions, decreasing the accumulation of amyloid in the brain of non-human primates with induced parkinsonism (Chen et al., 2015). The reduction in amyloid promoted by EGCG was observed in tissues of patients with PD (Xu et al., 2016), suggesting therapeutic potential. In addition, the neuroblastoma cells expressing wild-type or mutant α-syn were challenged by 6-hydroxydopamine (6-OHDA) and the genomic response was measured after EGCG treatment (Ma et al., 2010). The expression of α-syn sensitizes the cell to the insult and the effect of EGCG can be evaluated under the combination of genetic risk factors and environmental stress (simulated by 6-OHDA) that leads to oxidative damages similar to occurred in PD disease (Ma et al., 2010). The EGCG inhibited 70% of changes in the transcriptome induced by 6-OHDA, including the block of genes associated with erythroid-related nuclear factor 2 (Nrf2)-mediated antioxidant response (Ma et al., 2010). The knowledge about the modulation promoted by EGCG, an antioxidant, in stress response pathways may be used to understand the molecular bases of therapeutic strategies (Ma et al., 2010). Despite the 6-OHDA toxicity can be related to generation of ROS and both promote the caspase activation, it is important to identify the transcriptional network involved in neurotoxicity and EGCG action to search for new treatments (Ma et al., 2010).

### Effects of EGCG in Alzheimer’s Disease

Amyloid plaques and neurofibrillary tangles are hallmarks of AD. These structures are composed of amyloid-β peptide (Aβ) and tau protein, respectively (reviewed by Vaz and Silvestre, 2020). The Aβ formation and consequent aggregation depend on the sequential cleavage of amyloid precursor protein (APP) (Vaz and Silvestre, 2020). When APP is cleaved by α- and γ-secretase, the soluble product is non-amyloidogenic, but when cleavage occurs by β- and γ-secretase, amyloid-β is generated (Vaz and Silvestre, 2020). The Aβ is toxic and can aggregate, depositing in the brain tissue (Vaz and Silvestre, 2020). The new therapies focus on anti-amyloid compounds, increasing attention to the tau protein and aiming to act against the progression of the disease, not just alleviating symptoms (Vaz and Silvestre, 2020).

#### Indirect Effects of EGCG

The main green-tea polyphenol, EGCG, was first investigated for action against neuronal toxicity promoted by amyloid-β peptide with the focus on the antioxidant property of EGCG (Choi et al., 2001). The EGCG reduced the death of hippocampal neurons, and its protective effect was attributed to the scavenging of ROS (Choi et al., 2001). Furthermore, it has been reported that EGCG can restore nerve growth factor balance, reducing apoptosis and neurodegeneration through activation of the tropomyosin kinase A receptor (TrkA) (Liu et al., 2014). The EGCG neuroprotection also involved the nicotinic acetylcholine receptor α7 (nAChR α7) signaling cascade (Zhang X. et al., 2014). In rat neurons, EGCG protected against Aβ neurotoxicity by activating nAChR α7, which consequently activated phosphoinositide-3-kinase (PI3K), leading to Akt (protein kinase B) phosphorylation and attenuating the reduction of the anti-apoptotic Bcl-2 effector (Zhang X. et al., 2014). In a mouse model of AD, EGCG restored mitochondrial respiratory rates, adenosine triphosphate (ATP), and ROS levels and the membrane potential (Dragicevic et al., 2011). This investigation indicated that the EGCG action occurred in part by its antioxidant property and in part by stabilization of the electron transport chain (Dragicevic et al., 2011). The mitochondrial dysfunction can also be associated with prolonged exposure to oligomeric species of Aβ (He et al., 2011). These toxic species stimulate the ROS production that depends on the NADPH oxidase pathway and attenuate Ca^2+^ influx mediated by *N*-methyl-D-aspartate (NMDA)-receptor activity (He et al., 2011). The treatment with EGCG was able to protect against neurotoxic effects induced by Aβ oligomers, inhibiting ROS generation and mitigating mitochondrial damage (He et al., 2011). Moreover, the EGCG treatment can also prevented neuronal apoptosis induced by endoplasmic reticulum (ER) stress after Aβ exposure (Du et al., 2018). This array of mechanisms related to EGCG activity indicate a remarkably broad spectrum of molecular actions performed.

#### Activity of EGCG in APP Processing and Aβ Generation

It was found that EGCG can also suppress the increase in β-secretase expression (Shimmyo et al., 2008) and inhibit β-secretase activity directly (Jeon et al., 2003). Furthermore, in murine neuroblastoma cells transfected with the human APP mutant, EGCG inhibited the generation of Aβ_1__–__40_ and Aβ_1__–__42_ by increasing the action of α-secretase, which promotes the non-amyloidogenic processing of APP (Rezai-Zadeh et al., 2005).

Although the mechanism of action of EGCG has not yet been elucidated, metalloproteases and protein kinase C (PKC) may be involved. It was observed that EGCG depends on PKC and metalloproteinases for APP processing into soluble non-amyloidogenic products (Levites et al., 2003; Obregon et al., 2006). The increase in APP non-amyloidogenic processing promoted with EGCG treatment was attributed to activation of disintegrin and metalloproteinase domain-containing protein 10 (ADAM10) through estrogen receptor/phosphoinositide (Fernandez et al., 2010). Moreover, EGCG inhibited the activation of extracellular signal-regulated kinase (ERK) and the nuclear transcription factor-kB (NF-kB) induced by Aβ (Lee et al., 2009). Concomitantly, in AD mice, EGCG attenuated the reduction in α-secretase expression and the increase in β-secretase and Aβ that AD causes in the brain, suggesting that memory dysfunction was prevented by changes in APP processing (Lee et al., 2009). Thus, these changes in APP cleavage by secretases were correlated with the inactivation of ERK and NF-kB promoted by EGCG and the observations suggest that ERK and NF-kB may be modulating secretase activity (Lee et al., 2009). The EGCG also can decrease the Aβ levels by enhancing APP non-amyloidogenic processing when affecting c-Abl (Abelson tyrosine kinase) distribution in cells. The polyphenol can reduce nuclear translocation of c-Abl (Lin et al., 2009), involved in the regulation of cellular apoptosis (Yuan et al., 1997), and the interaction between c-Abl and FE65 (Lin et al., 2009), an adaptor protein involved in cellular movement and APP proteolytic processing (Wiley et al., 2007; Minopoli et al., 2012). In addition, it was demonstrated that EGCG can mitigate the expression of β-secretase and Aβ generation via nuclear peroxisome receptor activated by gamma receptor proliferator (PPARγ) (Zhang et al., 2017). Thus, reducing inflammatory agents, oxidative stress and apoptotic proteins (Zhang et al., 2017). The reduction in nuclear translocation of c-Abl inhibited glycogen synthase kinase-3β activity, an enzyme responsible for phosphorylating tau protein (Lin et al., 2009). Thus, the decrease in tyrosine phosphorylation of tau, which was indirectly generated by EGCG, can protect the cells (Lin et al., 2009).

#### EGCG Reduction in Aβ Levels and Amyloid Plaques

Analysis of the transgenic AD mouse model showed a reduction in brain amyloid plaques after treatment with EGCG and validated the results found in cells (Rezai-Zadeh et al., 2005). The reduction in amyloid plaques, Aβ levels and cognitive deficits was observed with intraperitoneal injection of EGCG (Rezai-Zadeh et al., 2005), as well as with oral administration in drinking water (Rezai-Zadeh et al., 2008). Furthermore, it was observed that the reduction in Aβ levels in mice treated with EGCG was accompanied by the inhibition in signaling to tumor necrosis factor alpha/c-Jun N-terminal kinase (TNF-α/JNK) and a decrease in insulin receptor substrate-1 (IRS-1), suggesting a correlation with the restoration of memory impairment by EGCG and the attenuation of insulin resistance (Jia et al., 2013).

Overall, it has been demonstrated that EGCG can reduce Aβ levels, inhibiting the deposition of plaques and recovering learning and memory functions that have been depleted by neurotoxic effects of aggregates (Chang et al., 2015; Schimidt et al., 2017; Mori et al., 2019; Bao et al., 2020). EGCG was effective in decreasing amyloid fibrillation (Wang et al., 2017; Rho et al., 2019), redirecting to non-toxic, amorphous species of oligomers (Ehrnhoefer et al., 2008; Sinha et al., 2012) and remodeling pre-formed fibrils (Ehrnhoefer et al., 2008; Palhano et al., 2013; Ahmed et al., 2017; Wang et al., 2017; Lee et al., 2020). However, the protective effects of EGCG in neurons are not restricted to the reduction of Aβ levels. It also protects against mitochondrial damage (Dragicevic et al., 2011), induced metal toxicity (Reznichenko et al., 2006; Hyung et al., 2013; Chan et al., 2016; Ayyalasomayajula et al., 2019), stress by ROS generation (Shimmyo et al., 2008; Kim et al., 2009; Ayyalasomayajula et al., 2019) and neuroinflammation events (Lee et al., 2009; Cheng-Chung Wei et al., 2016) related to AD. A study developed in transgenic *Caenorhabditis elegans* demonstrated that EGCG inhibited oligomerization and Aβ deposition, and in the worms exposed only to oxidative stress, the EGCG reduced the levels of small heat shock protein, under the control of DAF-2/insulin-like signaling pathway (Abbas and Wink, 2010). Thus, suggesting that EGCG can protect against age-related diseases, like AD, and ROS-mediated damages (Abbas and Wink, 2010).

#### EGCG Binding to β-Amyloid

To better understand the mechanism of EGCG binding to amyloid protein, thermodynamic analyses were performed (Wang et al., 2010). Hydrophobic interactions and hydrogen bonds appeared to be the main actors in the process of Aβ-EGCG interaction (Wang et al., 2010). There were gradual changes from hydrogen bonding to hydrophobic interactions during the increase in the EGCG/Aβ ratio and the experimental conditions as such increase in temperature, salt concentration or changes in pH facilitated the formation of the EGCG-protein bond (Wang et al., 2010). Hydrogen interactions have been shown to occur primarily in the protein backbone and hydrophobic interactions in the side-chains (Liu et al., 2011). In addition, it was found that van der Waals interactions and the participation of 12 amino-acid residues (F4, R5, F19, F20, E22, K28, G29, L34, M35, V36, G37, and I41) occurred during EGCG contacts with the Aβ peptide, preventing the conformation conversion of α-helices into β-sheets that is characteristic of Aβ_1__–__42_ (Liu et al., 2011). During analysis of Aβ_1__–__42_ fragments, it was observed that hydrogen bonds occur in Aβ_1__–__16_ more frequently, while hydrophobic interactions occur mainly in Aβ_17__–__42_. However, thermodynamic evaluations performed in different solutions containing the peptide fragments and EGCG did not suggest specific binding sites for EGCG (Wang et al., 2012). NMR characterizations of oligomers formed in the presence of EGCG showed that the compound interacts with the aromatic hydrophobic nucleus of Aβ (residues 17–20) (Lopez del Amo et al., 2012). There was an immobilization of 1–20 Aβ-peptide residues after EGCG interaction, inhibiting the characteristic β-sheet formation of amyloid aggregation (Lopez del Amo et al., 2012). Subsequent investigations demonstrated that 3 molecules of EGCG were attached to Aβ and the planar ring of EGCG prevented the β-sheets formation (Bleiholder et al., 2013). However, solid-state NMR assay indicated that the oligomers generated with EGCG treatment were not amorphous as previously described (Ehrnhoefer et al., 2008), but instead were well structured (Lopez del Amo et al., 2012). In the same study, it was reported that EGCG may have prevented the metal ions’ coordination with residues Y10, H13, and H14, justifying the loss of neurotoxicity by oligomers generated in the presence of EGCG (Lopez del Amo et al., 2012). This interaction with Y10 of Aβ peptide and the His influence was confirmed subsequently (Zhang B. et al., 2013). Furthermore, the metal-Aβ interaction was associated with neuronal toxicity and pathogenesis of AD, and evaluation of the effects of EGCG demonstrated that in the presence of metal that was free or complexed with the peptide, the induced neurotoxicity was reduced (Hyung et al., 2013). EGCG can bind to metal-Aβ species and also promote metal chelation, both related to this positive action of the compound (Hyung et al., 2013).

The first investigation of the binding between EGCG and the Aβ dimers (smallest aggregates) showed higher number of contacts of three aromatic rings of EGCG with Aβ and its preferential interaction with residues G29, A30, G37, G38, V39, and A42 of the backbone and strong with residues F4, F19, F20, T10, I31, I32, M35, V36, V39, and I41 of the side-chains (Zhang T. et al., 2013). Furthermore, analysis of the molecular mechanism of EGCG interactions during the remodeling of mature amyloid fibrils identified as the main factor the interactions between the compound and hydrophobic fiber sites (Palhano et al., 2013). This remodeling process depended on EGCG auto-oxidation, which generates a mixture of EGCG-quinone monomers and polymers (Palhano et al., 2013). The EGCG’s oxidized molecules formed Schiff bases with amyloid fibrils by reaction with free amines in the protein (Palhano et al., 2013). The crosslink thus generated was responsible for preventing fibril dissociation in toxic oligomeric species (Palhano et al., 2013). Despite the observation that oxidized EGCG was able to bind to fibrils, the role of EGCG auto-oxidation in driving the amyloid fibril remodeling remains unclear (Palhano et al., 2013). Through NMR spectroscopy analysis, it was observed that the flavon-3-ol unit of catechins was essential for the interaction with the Aβ oligomers and the EGCG interaction affinity can be enhanced by the presence of the gallate motif (Sironi et al., 2014). Later, it was observed that EGCG can interfere in the interaction of residues in the central region of Aβ (F19 and L34) that are important in the structure of amyloid fibrils (Tavanti et al., 2020). Molecular dynamics simulations indicated that EGCG alters the Aβ protofibril conformation by breaking the hydrogen bond between H6 and E11 residues, interacting with H14/Y10, and interacting with residue A42, disrupting the salt bridge with the side chain of K28 (Zhan et al., 2020). This same lysine also interacted with the gallic acid of EGCG, confirming that group’s critical role in protofibril disruption (Zhan et al., 2020). Thus, it was observed that the central interference promoted by EGCG in fibril formation is the disruption of inter-chain hydrogen bonds and salt bridges crucial to amyloid structure (Acharya et al., 2020). Even though the mechanism of action of EGCG has not been elucidated, the knowledge of some forms of interaction between the compound and amyloid proteins can generate valuable information for therapeutic strategies against aggregation. The EGCG seems to interact preferably with intrinsically disordered proteins as such Aβ and α-syn (Ehrnhoefer et al., 2008) and with flexible regions of ordered proteins (Fusco et al., 2018). The aromatic rings, hydroxyls groups and gallate motif of EGCG appear to be essential for the interactions with proteins and the anti-amyloid effectiveness (Rambold et al., 2008; Sironi et al., 2014; Fusco et al., 2018). Moreover, the hydrophobic interactions and hydrogen bonds represent the main form of protein-compound interaction, resulting in inhibition of the conversion in β amyloid toxic structures (Wang et al., 2010), and the break of saline bridges promoted by EGCG can be crucial to interrupt the fibril structuration (Acharya et al., 2020; Yao et al., 2020; Zhan et al., 2020).

## Drug Delivery Systems Containin Epigallocatechin-Gallate

Despite the several benefits promoted by EGCG treatment of AmD, their low intestinal absorbance and instability constitute an important limitation to consider in developing new therapeutic strategies (reviewed by Granja et al., 2017). Different types of nanocarriers have been evaluated for their ability to enhance EGCG efficacy, mainly related to this catechin’s antioxidant and anti-inflammatory properties (Granja et al., 2017). Nanolipid particles (lipid complexes: EGCG; formation of non-traditional micelles) have been synthesized to improve oral bioavailability of EGCG and BBB penetration and prevent APP cleavage of Aβ peptide by inducing α-secretase activity (Smith et al., 2010). The increased bioavailability of these particles may be important to reduce the required concentration of EGCG in promoting benefits and its future success in clinical trials (Smith et al., 2010). New types of nanoparticles containing EGCG have been evaluated for their ability to inhibit amyloid aggregation (Zhang J. et al., 2014; Debnath et al., 2016; Liu et al., 2017; Li et al., 2018; Singh et al., 2018; Fernandes et al., 2020). Selenium nanoparticles bound to EGCG and coated by the TET-1 peptide, which increases their delivery to neuronal cells, were effective at inhibiting amyloid cytotoxicity, blocking the Aβ aggregation and disaggregating mature fibrils (Zhang J. et al., 2014). These anti-amyloidogenic effects have also been shown during the evaluation of EGCG nanoparticles produced from polysuccinimide and functionalized with octadecylamine, dopamine and ethylenediamine and loaded with EGCG (Debnath et al., 2016). Enhancement of the chemical stability of EGCG by nanoparticle formation, their improvement in ease of cellular internalization, and stronger binding with amyloids were considered to contribute to their better performance (Debnath et al., 2016). In addition, when the EGCG was linked to negatively charged polymeric nanoparticles (NP10) it showed synergistic action against aggregation of Aβ and seeding capacity using a low concentration of the polyphenol (Liu et al., 2017). The NP10 inhibits the aggregation through hydrophobic binding and electrostatic repulsion and the hydrophobicity of EGCG stabilizes the Aβ in a stable oligomeric state, preventing the amyloid structuration (Liu et al., 2017). Both NP10 and EGCG, individually could act as anti-aggregating compounds, however in dual system were more efficient (Liu et al., 2017). In rats in which AD was induced by the administration of aluminum chloride, nanoparticles loaded with EGCG (synthesized by the solvent evaporation method in double emulsion) showed greater protective efficiency than free EGCG (Singh et al., 2018). These EGCG nanoparticles inhibited the accumulation of amyloid plaques and neurofibrillary tangles and reduced the immunoreactivity of the Aβ peptide and the ROS production in the brain tissues of AD rat model (Singh et al., 2018). Thus, the EGCG nanoparticles led to an increase in locomotor activity and recognition memory in these rats (Singh et al., 2018). In addition, EGCG together with ascorbic acid (AA) were encapsulated by a dual formulation of chemical polymers and also demonstrated a reduction in amyloid plaques and Aβ content in mice with familial AD (Cano et al., 2019). The increase in synapses and decrease in neuroinflammation generated after the treatment with encapsulated EGCG/AA were accompanied by improved learning and memory (Cano et al., 2019). The AA was responsible for creating an antioxidative environment for EGCG and promoted an increase in the positive effects of nanoparticles (Cano et al., 2019). In the mouse model with PD, similar results were observed when nanolipid particles carrying EGCG improved motor performance, decreased α-syn aggregation in neurons and protected dopaminergic cells (Li et al., 2018). These nanoparticles were more permeable and accumulated in brain tissue because of the link with the B6 peptide, which has a high affinity for the transferrin endothelial receptor, and also because of the loading with supermagnetic iron oxide nanocubes (Li et al., 2018). Furthermore, liposomes assembled with 1-palmitoyl-2-oleoyl-sn-glycero-3-phosphate and leptin used for EGCG delivery were more permeable than unmodified liposomes, reducing MPTP toxin-induced neurotoxicity and the cellular expression of α-syn and proteins involved in apoptotic processes (Kuo et al., 2021). Finally, a different concept was introduced by the synthesis of functional spheres of EGCG, synthesized by a simple method of catechin auto-oxidation under controlled heating and in the presence of a specific metal concentration (Chen et al., 2013). These functional microparticles, with oxidized EGCG as carrier, have been shown to inhibit α-syn aggregation, reduce the cytotoxicity of oligomers and, modestly, remodel mature fibrils (Fernandes et al., 2020). Moreover, when the EGCG microparticle was loaded with an additional amyloidogenesis inhibitor, ortho-iminoquinone (Largeron and Fleury, 2012; Fernandes et al., 2017), its activity increased, demonstrating a synergistic action between the microcarrier and the loaded molecule (Fernandes et al., 2020).

## Clinical Trials Involving Green Tea and EGCG

Since green tea components are well absorbed and bioavailable in humans (Nakagawa et al., 1997; van het Hof et al., 1998; Chow et al., 2003), several clinical trials have been conducted to dissect the role of complete green tea or its purified constituents in different conditions and diseases.

In human clinical trials, green tea extracts and EGCG were shown to be safe for use in children and adults, including during prolonged periods (Kumar et al., 2016; de la Torre et al., 2020; Vilela et al., 2020), which reinforces the treatment potential of those components.

Most clinical trials involving green tea and EGCG are related to several cancer types, cardiovascular diseases, and metabolic disorders such as diabetes, dyslipidemia and obesity. Despite evidence from *in vitro* and *in vivo* studies involving amyloidoses and EGCG, only a few clinical trials are registered, and even fewer have produced accessible results. In phase 1 clinical trials, green tea reduced cardiac TTR amyloidosis-related symptoms and amyloid plaques (Kristen et al., 2012; Aus dem Siepen et al., 2015) and improved health quality in patients.

Neurological clinical trials are almost entirely restricted to cognitive performance studies. In a study conducted in 2012, EGCG (300 mg) improved neurological effects during alpha, beta, and theta brainwave stimulation, promoting, among other effects, increased calmness and reduced stress self-evaluated by healthy individuals over periods of 120 min (Scholey et al., 2012). These studies are corroborated by a more recent phase 1 trial conducted by de la Torre et al. (2020). EGCG improved cognition and functional competence when combined with cognitive training during a 3-month follow-up (de la Torre et al., 2020).

Epigallocatechin-gallate also abrogated cognitive deficits related to Down syndrome, an amyloid-related disease (reviewed by Abrahamson et al., 2019), since its administration during 3 or 12 months induced episodic memory and working memory improvement and visual recognition memory and adaptive behavior, respectively (de la Torre et al., 2014, 2016). The consumption of green tea was related to a reduced risk of dementia n elderly Japanese (Tomata et al., 2016), suggesting that its use may be related to a better prognosis in AD.

In patients with multiple system atrophy, a disease related to α-synuclein aggregation, EGCG administered daily for 48 weeks showed no effect against disease progression (Levin et al., 2019).

As reviewed here, green tea EGCG is a potent molecule with several therapeutic properties against different neurological diseases. However, there is a lack of clinical trials involving this promising molecule against these types of disease and amyloidoses especially.

## Final Considerations

**FIGURE 2:**
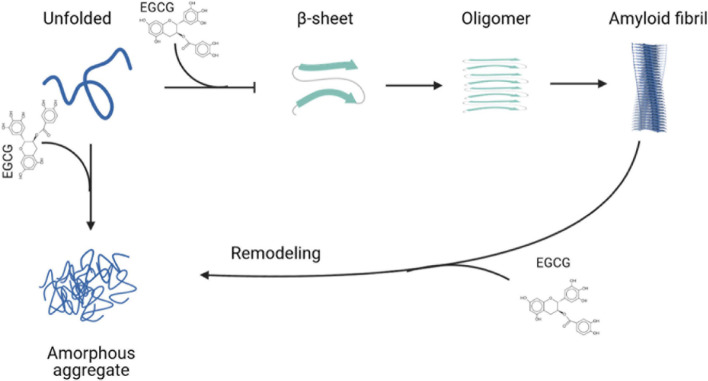
Epigallocatechin-gallate (EGCG) interferes in different steps of amyloid formation. EGCG can bind to unfolded monomers and inhibit the formation of amyloid fibrils or amorphous aggregates. It also is able to remodel amyloid fibrils to form amorphous aggregates. Created with BioRender.com.

Considering the evidence presented above, the use of EGCG in amyloidogenic neurodegenerative diseases is a very promising therapeutic tool, since it has been used in pre-clinical and clinical studies to treat several amyloidoses. Figure 2 summarizes the interference and anti-amyloid effects of EGCG in different steps of protein aggregation and amyloid formation.

## Author Contributions

LF and TRCP both wrote the manuscript. DF and FLP edited, approved, and finalized the manuscript.

## Conflict of Interest

The authors declare that the research was conducted in the absence of any commercial or financial relationships that could be construed as a potential conflict of interest.

## Publisher’s Note

All claims expressed in this article are solely those of the authors and do not necessarily represent those of their affiliated organizations, or those of the publisher, the editors and the reviewers. Any product that may be evaluated in this article, or claim that may be made by its manufacturer, is not guaranteed or endorsed by the publisher.
